# Estimated Tubular Secretion of Creatinine and Risk of Kidney Failure: The AASK Trial

**DOI:** 10.1016/j.xkme.2026.101433

**Published:** 2026-06-15

**Authors:** Pranav S. Garimella, Kevin M. Cummins, Jennifer Gassman, Francis Gabbai, Andrew S. Levey, Joachim H. Ix

**Affiliations:** 1 Division of Nephrology and Hypertension, Department of Medicine, University of California San Diego, La Jolla, CA; 2 Division of Global Public Health, University of California San Diego, La Jolla, CA; 3 Department of Quantitative Health Sciences, Cleveland Clinic, Cleveland, OH; 4 Nephrology Section, Veterans Affairs San Diego Healthcare System, La Jolla, CA; 5 Division of Nephrology, Tufts Medical Center, Boston, MA

To the Editor:

Kidney tubular secretion is an important nonglomerular kidney function and is critical for excretion of toxins and many medications.^1^^,^^2^ Creatinine is freely filtered by the glomerulus and actively secreted by the proximal tubule. Therefore, 24-hour creatinine clearance (CrCl) overestimates the glomerular filtration rate (GFR) by 10%-20%. In studies that have measured GFR (mGFR) using an isotope such as iohexol or iothalamate, and CrCl, the measured tubular secretion of creatinine (mTSCr) can be calculated as the difference between CrCl and mGFR. In other studies, we have previously demonstrated that lower mTSCr is associated with incident kidney failure with replacement therapy (KFRT), independent of mGFR, proteinuria, or other risk factors.^3^ However, measuring GFR is technically challenging and is often unavailable in clinical settings. Updated equations to estimate GFR (eGFR) are accurate and are routinely done as part of clinical care and are universally applicable to all populations irrespective of race.^4^ We hypothesized that the estimated tubular secretion of creatinine (eTSCr) obtained from the difference between CrCl and eGFR would provide a reliable estimate of mTSCr and would similarly associate with clinical outcomes. If demonstrated, this would allow for easier assessment of tubular secretion and its impact on clinical outcomes in large epidemiological studies as only eGFR and measured CrCl would be required.

We conducted a retrospective analysis of all participants enrolled in the African American Study of Kidney Disease, which was a randomized trial to investigate the effects of blood pressure control and the use of specific antihypertensive regimens on the progression of chronic kidney disease.^5^ The study was deemed institutional review board exempt by the UC San Diego Institutional Review Board (#191465XX) and informed consent was not required. This prospective analysis included 999 participants with baseline measures of iothalamate mGFR, creatinine-based eGFR, and 24-hour urine CrCl. The first 2 values for mGFR, 24-hour CrCl, and eGFR taken 6 months apart were averaged to improve accuracy and reduce the influence of bias due to measurement error. Tubular secretion of creatinine was calculated as the difference between the following: (1) CrCl and mGFR (mTSCr) and (2) CrCl and eGFR (eTSCr). The primary outcome was KFRT and secondary outcomes were cardiovascular disease (CVD) and all-cause mortality. Cox proportional hazards regression was used to evaluate the association of TSCr with these outcomes after adjustment for age, self-reported sex, body mass index, randomization arm, smoking, prevalent CVD, systolic blood pressure, antihypertensive medications, mGFR, and protein-creatinine ratio.

**Figure 1:**
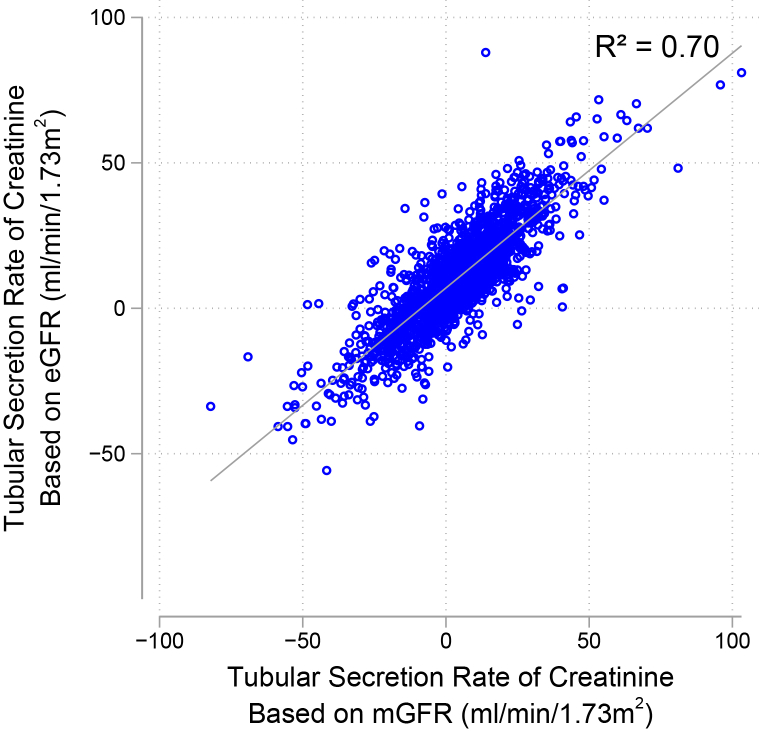
Correlation between mTSCr and eTSCr. The figure shows the correlation between mTSCr obtained from iohexol-measured GFR and 24-hour urine--measured creatinine clearance and eTSCr obtained from estimated GFR and 24-hour urine--measured creatinine clearance. The line is the ordinary least squares regression line (eTSCr = 7.00 + mTSCr × 0.81). Abbreviations: eGFR, estimated glomerular filtration rate; eTSCr, tubular secretion of creatinine using eGFR; mGFR, measured glomerular filtration rate; mTSCr, tubular secretion of creatinine using mGFR.

The mean age of the cohort was 54 years, and 38% were women. At baseline, the mean mGFR was 45.3 mL/min/1.73 m^2^, and the mean CrCl was 49.3 mL/min (Table 1). Mean mTSCr and eTSCr were 4.1 and 6.5 mL/min/1.73 m^2^, respectively, and both were strongly correlated (r = 0.84) with each other (Fig 1). During a mean of 4.2 years of follow-up, there were 149 KFRT, 82 all-cause mortality, and 132 incident CVD events. In continuous models, each 10-mL higher mTSCr was associated with 27% lower risk of KFRT (hazard ratio 0.73; 95% confidence interval 0.58-0.93) and each 10-mL higher eTSCr was associated with 39% lower risk of KFRT (hazard ratio 0.61; 95% confidence interval 0.45-0.81), after adjustment for GFR, proteinuria, and other potential confounding factors. Neither mTSCr nor eTSCr was found to be associated with lower risk of all-cause mortality or CVD events (*P* > 0.16 for both, Tables S1 and S2).

**Table 1:** Baseline AASK Trial Participant Characteristics Across mTSCr (CrCl-mGFR) Quartiles

Quartile mTSCr Range, mL/min/1.73 m^2^	ALL -33.15, 72.67	Q1 -33.15, 2.17	Q2 2.20, 9.78	Q3 9.80, 18.08	Q4 18.22, 72.67
n	999	250	250	250	249
Age, y	54.3 (10.51)	53.75 (10.84)	53.8 (11.18)	54.22 (10.15)	55.43 (9.76)
Males	62%	58%	59%	62%	67%
BMI, kg/m^2^	30.63 (6.57)	29.88 (6.76)	30.2 (7.13)	30.53 (6.15)	31.9 (6.04)
History of CVD or HF	51%	52%	54%	52%	47%
Smoking status					
Never smoker	42%	39%	44%	38%	49%
Former smoker	29%	37%	30%	29%	22%
Current smoker	28%	24%	27%	34%	29%
mGFR, mL/min/1.73 m^2^	45.31 (16.38)	46.84 (15.8)	37.8 (16.31)	42.22 (15.66)	54.41 (12.79)
CrCl, mL/min/1.73 m^2^	49.36 (20.98)	36.15 (14.68)	39.93 (16.21)	49.42 (15.82)	72.04 (16.06)
mTSCr, mL/min/1.73 m^2^	4.05 (14.03)	-10.69 (12.1)	2.12 (6.76)	7.2 (7.57)	17.63 (11.13)
eTSCr, mL/min/1.73 m^2^	10.22 (14.07)	--6.86 (8.36)	6.01 (2.15)	13.75 (2.36)	28.08 (8.48)
Urinary protein-creatinine ratio, mg/g	0.3 (0.49)	0.22 (0.36)	0.43 (0.6)	0.38 (0.55)	0.19 (0.39)
Systolic BP, mm Hg	149.79 (23.71)	151.07 (24.77)	152.61 (23.86)	148.08 (21.56)	147.39 (24.27)
Intensive BP control arm	50%	46%	51%	50%	52%
Randomized to ACE inhibitors	40%	37%	39%	41%	43%
Randomized to beta blockers	40%	42%	44%	41%	34%
Randomized to calcium channel blockers	20%	21%	17%	18%	22%
Total cholesterol, mg/dL	211.58 (44.54)	205.79 (46.62)	214.62 (41.83)	211.28 (43.73)	214.68 (45.45)
LDL cholesterol, mg/dL	136.26 (40.31)	130.37 (44.16)	136.86 (38.45)	135.62 (39.28)	143.02 (37.95)
HDL cholesterol, mg/dL	48.44 (16.12)	50.32 (16.43)	48.83 (16.36)	48.02 (15.18)	46.62 (16.33)
Triglycerides, mg/dL	137.51 (70.06)	131.37 (63.73)	142.27 (84.84)	138.5 (68.62)	138.52 (61.6)
Years of follow-up	4.2 (1.43)	4.19 (1.38)	3.93 (1.49)	4.23 (1.43)	4.44 (1.38)

*Note*: Cell values are count, mean (standard deviation), or percentage.

Abbreviations: ACE, angiotensin-converting enzyme; BMI, body mass index; BP, blood pressure; CrCl, creatinine clearance; CVD, cardiovascular disease; eTSCr, tubular secretion of creatinine using estimated glomerular filtration rate; HDL, high-density lipoprotein; HF, heart failure; LDL, low-density lipoprotein; mGFR, measured glomerular filtration rate; mTSCr, tubular secretion of creatinine using mGFR.

In this analysis of data from randomized, controlled African American trial participants with hypertension and chronic kidney disease and concurrent 24-hour urine CrCl and mGFR measurements, higher mTSCr and the easier to obtain eTSCr were highly correlated with one another and were both associated with lower risk of incident KFRT after adjusting for multiple confounding variables, including baseline GFR. Neither mTSCr nor eTSCr was detectably associated with CVD or mortality. These data are supportive of prior findings and additionally indicate that eTSCr can be used as a surrogate of mTSCr. We found the association of eTSCr (hazard ratio 0.61) to be stronger with KFRT compared with mTSCr (hazard ratio 0.73). We believe this observed difference is attributable to differences in measurement precision because mGFR is susceptible to diurnal variation, hemodynamic shifts, and collection timing errors. Prior analyses in the Chronic Renal Insufficiency Cohort study have similarly demonstrated that the higher random error inherent in mGFR protocols can attenuate associations with clinical outcomes compared with creatinine-based estimates.^6^

The last decade has seen the development^7^ and validation of several endogenous proximal tubular secretory markers, which are primarily cleared by organic anion transporters. Although these solutes can identify the risk for kidney failure, acute kidney injury, and adverse effects of drugs,^8^ assays for these solutes are only available in research laboratories with mass spectrometry capabilities. Creatinine, on the other hand, provides an index of secretion through the organic cation transporters and provides a reliable and readily measurable alternative to estimate tubular secretion.^3^^,^^9^ These tools may allow for a more complete assessment of overall kidney function, beyond glomerular filtration and proteinuria alone.^10^

Estimating tubular secretion using eGFR instead of mGFR offers several advantages in clinical practice and research. The accessibility and cost-effectiveness of eGFR calculations, which rely on serum creatinine, are widely available, making them more practical for routine use and large-scale studies. The requirement of a single blood test facilitates implementation across various health care settings, enables the standardization of eGFR formulas, such as the CKD-EPI (Chronic Kidney Disease Epidemiology Collaboration) equations, and allows for better comparability of results. Estimation of TSCr using eGFR (eTSCr) still requires timed urine collection for CrCl, but this is easier to implement than mGFR using exogenous tracers.^9^ Two important limitations of our study are the lack of ability to generalize the results, given that all participants included in the African American Study of Kidney Disease trial identified as African American, and the absence of information on medicines that competitively inhibit tubular secretion, which could influence creatinine handling.

In summary, we validate prior findings that low mTSCr is associated with kidney failure independent of mGFR, proteinuria, and other risk factors in a large cohort of African American patients enrolled in a trial of hypertension management. In addition, we demonstrate that estimating TSCr using eGFR provides a robust alternative for mTSCr that is highly correlated, provides similar associations with clinical endpoints, and may be easier to obtain in clinical and research settings than mTSCr.
