# Supplementary material for: Estimated Tubular Secretion of Creatinine and Risk of Kidney Failure: The AASK Trial
Source: Kidney Med. 2026 Jun 15;8(8):101433. doi: 10.1016/j.xkme.2026.101433 (PMC13416659; doi:10.1016/j.xkme.2026.101433)
Supplement: Supplementary File (PDF) — Tables S1 and S2. [file mmc1.pdf]

Table S1: Association of mTSCr with end-stage kidney disease, mortality, and cardiovascular disease events

mTSCr	Events (N)	HR, 95% CI Model 1	HR, 95% CI Model 2	HR, 95% CI Model 3	HR, 95% CI Model 4
	**End-Stage Kidney Disease**				
**Per 10ml higher**	149	0.9 (0.8, 1), 0.055	0.92 (0.82, 1.03), 0.144	0.93 (0.83, 1.04), 0.194	**0.73 (0.58, 0.93), 0.012**
**Q1**	29	3.17 (1.6, 6.29), 0.001	3.01 (1.51, 5.98), 0.002	2.97 (1.49, 5.91), 0.002	1.77 (0.86, 3.61), 0.119
**Q2**	66	5.73 (3.01, 10.92), 0	5.48 (2.86, 10.47), 0	5.66 (2.95, 10.86), 0	1.04 (0.52, 2.09), 0.915
**Q3**	41	4.69 (2.44, 9.04), 0	4.72 (2.45, 9.11), 0	5 (2.59, 9.68), 0	0.76 (0.37, 1.54), 0.443
**Q4**	12	Ref	Ref	Ref	Ref
	**ALL CAUSE MORTALITY**				
**Per 10ml higher**	82	0.9 (0.77, 1.05), 0.175	0.9 (0.76, 1.05), 0.18	0.94 (0.79, 1.1), 0.42	0.92 (0.77, 1.1), 0.389
**Q1**	25	1.64 (0.88, 3.07), 0.122	1.59 (0.85, 2.99), 0.15	1.35 (0.71, 2.57), 0.353	1.22 (0.64, 2.31), 0.553
**Q2**	21	1.26 (0.66, 2.41), 0.491	1.16 (0.6, 2.23), 0.658	1.06 (0.55, 2.05), 0.861	0.78 (0.39, 1.56), 0.487
**Q3**	20	1.23 (0.64, 2.37), 0.536	1.19 (0.62, 2.31), 0.597	1.14 (0.59, 2.23), 0.693	0.87 (0.44, 1.72), 0.682
**Q4**	16	Ref	Ref	Ref	Ref
	**CVD EVENTS**				
**Per 10ml higher**	132	0.98 (0.87, 1.1), 0.716	0.96 (0.85, 1.09), 0.532	1.01 (0.89, 1.14), 0.911	1 (0.88, 1.14), 0.953
**Q1**	36	1.27 (0.76, 2.12), 0.36	1.35 (0.81, 2.26), 0.256	1.17 (0.69, 1.97), 0.555	1.16 (0.69, 1.95), 0.586
**Q2**	39	1.58 (0.97, 2.59), 0.067	1.66 (1.01, 2.72), 0.044	1.48 (0.9, 2.44), 0.125	1.4 (0.84, 2.34), 0.19
**Q3**	32	1.35 (0.81, 2.23), 0.249	1.4 (0.84, 2.32), 0.194	1.31 (0.78, 2.18), 0.306	1.18 (0.7, 1.99), 0.544
**Q4**	24	Ref	Ref	Ref	Ref

Model 1: unadjusted, Model 2: adjusted for age, sex, BMI, randomization arm, Model 3: Model 2+ smoking, CVD, systolic BP, antihypertensive medications
Model 4: Model 3+ urine protein/creatinine + baseline mGFR
mTScr: Tubular secretion of creatinine using measured glomerular filtration rate (mGFR)

Table S2: Association of eTSCr with end-stage kidney disease, mortality and cardiovascular disease events

eTSCr	Events (N)	HR, 95% CI Model 1	HR, 95% CI Model 2	HR, 95% CI Model 3	HR, 95% CI Model 5
	**ESKD**				
**Per 10ml higher**	149	0.81 (0.72, 0.91), <0.001	0.83 (0.74, 0.93), 0.001	0.83 (0.74, 0.93), 0.001	0.61 (0.45, 0.81), 0.001
Q1	35	3.86 (1.91, 7.8), <.001	3.72 (1.84, 7.53), <.001	3.69 (1.82, 7.49), <.001	0.61 (0.28, 1.29), 0.194
Q2	67	7.88 (4.06, 15.33), <.001	7.66 (3.92, 14.95), <.001	8.11 (4.14, 15.89), <.001	0.52 (0.25, 1.09), 0.083
Q3	37	3.85 (1.91, 7.74), <.001	3.76 (1.87, 7.59), <.001	3.87 (1.92, 7.82), <.001	0.45 (0.22, 0.93), 0.032
Per 10ml higher	149	0.81 (0.72, 0.91), <.001	0.83 (0.74, 0.93), 0.001	0.83 (0.74, 0.93), 0.001	0.61 (0.45, 0.81), 0.001
	**ALL CAUSE MORTALITY**				
Per 10ml higher	82	0.84 (0.72, 0.98), 0.031	0.84 (0.72, 0.99), 0.039	0.87 (0.74, 1.03), 0.10	0.89 (0.74, 1.06), 0.194
Q1	31	2.06 (1.13, 3.77), 0.019	1.99 (1.08, 3.67), 0.028	1.71 (0.92, 3.18), 0.091	1.2 (0.62, 2.32), 0.589
Q2	16	1.03 (0.51, 2.05), 0.941	1.01 (0.5, 2.03), 0.983	0.91 (0.44, 1.84), 0.784	0.50 (0.23, 1.10), 0.083
Q3	19	1.15 (0.59, 2.23), 0.684	1.08 (0.55, 2.11), 0.821	0.99 (0.5, 1.96), 0.978	0.72 (0.36, 1.45), 0.357
Q4	16	Ref	Ref	Ref	Ref
	**CVD EVENTS**				
**Per 10ml higher**	132	1.02 (0.91, 1.15), 0.695	1.01 (0.89, 1.13), 0.934	1.06 (0.94, 1.20), 0.344	1.08 (0.95, 1.24), 0.223
**Q1**	32	1.00 (0.62, 1.62), 0.991	1.06 (0.65, 1.72), 0.822	0.87 (0.53, 1.43), 0.587	0.80 (0.48, 1.33), 0.389
**Q2**	32	1.08 (0.67, 1.75), 0.758	1.15 (0.71, 1.87), 0.577	0.99 (0.60, 1.62), 0.964	0.86 (0.51, 1.46), 0.578
**Q3**	34	1.05 (0.65, 1.69), 0.830	1.11 (0.69, 1.79), 0.664	0.99 (0.61, 1.61), 0.978	0.88 (0.54, 1.45), 0.625
**Q4**	34	Ref	Ref	Ref	Ref

Model 1: unadjusted, Model 2: adjusted for age, sex, BMI, randomization arm, Model 3: Model 2+ smoking, CVD, systolic BP, antihypertensive medications
Model 4: Model 3+ urine protein/creatinine + baseline eGFR
eTScr: Tubular secretion of creatinine using estimated glomerular filtration rate (eGFR)
